# “I tell you what it’s the hardest job”: the experiences of family carers providing support for people with dementia at home in the last year of life

**DOI:** 10.1186/s12877-025-06719-6

**Published:** 2025-12-06

**Authors:** Caroline Mogan, Karen Harrison Dening, Christopher Dowrick, Kelly McCarrick, Mari Lloyd-Williams

**Affiliations:** 1 Nursing and Health, Faculty of Health, Liverpool John Moores University, 79 Tithebarn Street, Liverpool, L2 2ER UK; 2 Dementia UK, One Aldgate, London, EC3N 1RE UK; 3 Primary Care and Mental Health, University of Liverpool, Waterhouse Building Block B 1 st Floor 1-5 Brownlow Street, Liverpool, L69 3GL UK; 4 Cheshire and Wirral Partnership NHS Trust Research Department, Sycamore House Ellesmere, Ellesmere Port, CH65 9HQ UK

**Keywords:** Dementia, End-of life, Palliative care, Home death, Informal care, Family carers, Qualitative study

## Abstract

**Background:**

More people are dying at home with dementia. While there is growing recognition of the central role that family carers play when supporting people with dementia to die at home, knowledge gaps remain around how to best support them as they care for the person during the last year of life.

**Aim:**

To explore the experiences of bereaved family carers who had provided care for a person with dementia living at home in the last year of life.

**Design:**

A descriptive qualitative study based on a constructivist epistemology using in-depth semi-structured interviews. Data were analysed using reflexive thematic analysis.

**Participants:**

Twenty-nine bereaved family carers who had supported a person with dementia living at home in the last year of life.

**Results:**

Caring for a person with dementia at home in the last year of life can be emotionally, mentally, and physically overwhelming. Family carers described the challenges they faced when trying to ensure that they met the needs of the person with dementia so that they could remain at home. Three overarching themes were developed from the data: Managing end-of-life symptoms and associated conditions; Living with uncertainty; and Impacts on wellbeing.

**Conclusions:**

Supporting a person with dementia at home in the last year of life can jeopardise family carers’ own health, finances, relationships, and overall wellbeing. Many felt that they had limited understanding about the prognosis of dementia and how this would impact on caring at home, leaving them feeling unsupported amidst the incredible responsibilities placed on them. Family carers would benefit from training on how to provide practical aspects care for the person with dementia in the home, as well as support when making decisions for the person with dementia towards the end-of-life.

## Background

Dementia is a growing global health challenge that affects millions of individuals and their families. As life expectancy increases worldwide, the prevalence of dementia has also increased, placing significant pressure on health and social care systems, carers, and communities [1]. Approximately 50 million people are currently living with dementia worldwide, and this number is projected to reach 152 million people by 2050 [2].

In the UK, it is estimated that there are 982,000 people living with dementia, and this is projected to rise to 1.6 million by 2050 [3]. Dementia is a life-limiting condition that is associated with a high symptom burden, particularly in the advanced stages [4]. Many people with dementia die of a medical complication, such as pneumonia or another infection, but dementia itself can be the cause of death, for example, through frailty, malnutrition, and dehydration when a person with dementia can no longer eat safely and move independently [5]. Dementia is the leading cause of death in England and Wales, and in 2024 accounted for a total of with 68,273 deaths (12.1% of all deaths) [6].

By 2040, it is estimated that 220 000 people will die each year with dementia in England and Wales [7]. There are several unique challenges in providing palliative and end-of-life care to people with dementia. These include communication difficulties, dementia not being recognised as a terminal condition, discontinuity of care and lack of coordination across care settings, and elements of uncertainty in several areas including patient wishes and prognosis [8].

More people are dying at home from dementia than ever before. In 2020 there were an excess of 2,095 deaths from dementia in private homes, in England. This is a rise of 79% compared with the average recorded for the same period over the past five years [9]. Patient preferences [10, 11] and government policies [12], indicate the importance of increasing end-of-life care at home and reducing deaths in hospital. However, for a person with dementia to be able to die at home, there is heavy reliance on the contributions of unpaid, informal care [13].

In this paper, the term “family carer” is used to encompass all informal carers (i.e., family and friends/neighbours) who provide support to a person with dementia. This is the term that previous studies have found that carers supported most, with “carer” preferred to “caregiver” and “family” preferred to “informal” (even given that this group may include non-family members such as neighbours and friends) in distinguishing family carers from “paid” or “formal” carers [14].

In the UK, there are approximately 700,000 family carers [15] who collectively provide 1.3 billion hours of unpaid care a year [16] saving the UK economy GBP 13.9 billion [17]. While some report positive aspects of caring for a person with dementia such as satisfaction [18] and personal growth [19, 20], there is a substantial body of evidence exploring the negative impacts. Family carers often report a significant sense of burden resulting in poorer physical and psychological wellbeing [21–24], which is perceived to increase as the condition progresses and the person with dementia reaches the end-of-life [25]. Female carers are especially at risk of depression, and around two-thirds of carers for people with dementia are women [26]. Additionally, the majority of family carers report that they have had no, or insufficient, support [26] and suggest that caring for a person with dementia is more impactful than with other health conditions [27].

The marked increase in dementia deaths in private homes has notable implications for family carers. While there is growing recognition of the central role that family carers play when supporting people with dementia to die at home, knowledge gaps remain around how to best support those who are caring for a person with dementia during the last year of life [13]. There is also a need to better understand how the home setting for end-of-life care impacts people with dementia and their carers.

The aim of this study was to explore the experiences of bereaved family carers who had supported a person with dementia at home in the last year of life. Other findings from this study that focussed on family carer perceptions of health and social care services for people with dementia at home in the last year of life have been reported elsewhere [28].

## Methods

### Design and sampling

This was a descriptive qualitative study based on a constructivist epistemology using in-depth semi-structured interviews. Interviews aimed to explore participants’ experiences of caring for a person with dementia at home at the end-of-life. The constructivist lens allowed for contextual understanding of family carers’ experiences by paying close attention to the social, cultural, and historical factors that influenced their perspectives.

Using purposive sampling techniques, bereaved family carers who had supported someone with dementia at home during their last year of life were recruited. Due to the sensitive nature of the topic and the potential for distress, those bereaved within the last three months were excluded from participation. Additionally, those who were bereaved longer than three years were also excluded as it was felt that this may affect recall. While there is no fixed guidance on when to include those who have been bereaved to research, the evidence suggests that these were appropriate parameters [29, 30].

A broad approach to recruitment was used. Appeals for participants were made via posters displayed in public venues (e.g. GP practices and community halls) across two areas in the North-West of England (Cheshire and Wirral) and in some parts of North Wales. These areas are socio-demographically diverse and range from dense urban/industrial areas to semi-rural and rural areas. Potential participants could then contact the researcher either by telephone or email to express their intertest in taking part. Additionally, the National Institute for Health Research’s “Join Dementia Research” was utilised, which is an online self-registration service that enables volunteers to register their interest in taking part in dementia research.

Participants were also identified and approached by clinicians known to them from local Memory Services. After being given an information pack, the participant could then contact the research team directly or return a reply slip to express an interest in taking part.

### Data collection

Interviews were conducted between November 2017 and June 2018 by one of the authors (C.M.), an experienced dementia researcher. An interview schedule was developed (Appendix 1), informed by an earlier review [13], and the research team, consisting of clinicians and researchers in primary care, dementia care, and palliative care. Initial interview questions were kept open and general, allowing participants to reflect upon their whole experience of caring for the person with dementia, how this developed over time and explore their relationship. Sub questions were asked about the different types of support they received, and then more specifically about the end-of-life phase. Questions derived from the literature specifically about symptom management were also asked. Additional questions explored why the carer felt it was important for the person with dementia to stay at home at the end-of-life. The final section considered the main challenges and facilitators of caring for a person with dementia at home at the end-of-life. The interview schedule was iteratively modified throughout the data collection period to ensure follow-up with categories in subsequent interviews.

All interviews were audio-recorded. Most interviews took place in the participants own homes, often where the person with dementia had died. This allowed the researcher to observe the living environment including the physical challenges that the family carers faced when caring at home. This provided additional context to participant experiences and helped to inform the interpretation of the data. Two interviews took place on university premises and one via Skype. Extensive field notes were taken following each interview.

### Data analysis

Interviews were transcribed *verbatim*, anonymised, and imported into NVivo 11 (QSR International (UK) Ltd) to organise data. Reflexive thematic analysis was used to analyse data and develop themes [31]. Transcripts were read and re-read by two members of the research team (CM and MLW). CM coded the data inductively for themes relevant to participants’ experiences of caring for a person with dementia at home. A coding frame was developed and checked against the data to ensure fit. 10% of transcripts were double coded by another researcher (M.L.W.). Through a series of discussions with the whole team (experienced in dementia care, primary care, and palliative care), the coding framework was reviewed, and related codes were grouped into themes. Themes were refined and finalised through further discussion, and authors agreed with the final analysis, interpretation, and reporting.

### Ethical considerations

Participants were provided with oral and written information about the study, and all provided written informed consent. They were informed they could stop the interview at any time due to its sensitive topic, however, none expressed a wish to stop an interview or required additional support. Though research participants affected by serious illness can find research interviews to be a positive experience [32, 33], participants were provided with information about support organisations as part of the study debrief.

Ethical approvals were obtained from University of Liverpool Central University Research Ethics Committee [Ref: 1392].

## Results

Twenty-six interviews were conducted with twenty-nine bereaved caregivers. This included twenty-three individual interviews, and three interviews with two participants, who asked to be interviewed together. Sixteen participants were recruited from Memory Services, eight responded to posters in public venues, and two were recruited through “Join Dementia Research”. Most participants were female (*n* = 23) and twelve of whom were daughters of the person with dementia. All described their ethnicity as “White British”. Sample characteristics are reported in Table 1..

**Table 1:** Characteristics of Carer and Person with Dementia

Variable	*N*	Variable	*N*
Gender of carer		Relationship to Person with Dementia	
Female	23	Spouse/Partner	9
Male	6	Female (n=6)	
Age of Carer		Male (n=3)	
20–29	1	Adult Child	14
30–39	0	Son (n=2)	
40–49	2	Daughter (n=12)	
50–59	7	Granddaughter	1
60–69	10	Niece	3
70–79	5	Friend	2
80–89	4	Female (n=1)	
Age of person with dementia at death		Male (n=1)	
60–69	2	Years Spent Caring	
70–79	5	0–5	16
80–89	11	6–10	11
90–99	8	11–15	2
		Place of Death	
		Home	16
		Hospital	2
		Care Home	7
		Hospice	1

The data below represents carers’ experience of providing care to a person with dementia at home at the end-of-life. Three overarching themes were developed: managing end-of-life symptoms and associated conditions, living with uncertainty and impacts on wellbeing.

## Managing end-of-life symptoms and associated conditions

Family carers reported that in the last few months of life the person with dementia had become fully dependent on others for all care. The most common symptoms and conditions that family carers were expected to manage were incontinence, frailty and falls, pain, and dysphagia.

### Incontinence

Family carers perceived continence management as one of the most difficult aspects of caring at home towards the end-of-life. It was viewed as both tiring and upsetting, and carers expressed concern about whether they had managed it appropriately. They reported difficulties in finding advice and information about incontinence, and believed that products supplied by the National Health Service (NHS) were ineffective due to their poor quality:

*All I was doing was washing*,* I’d wash and then dry*,* get all of the bed made nice*,* and we’d use the pads*,* then they didn’t work*,* I’ll tell you that’s the hardest part of it*,* is getting up in the night getting him cleaned up*,* every night he’d wet the bed*,* now and again he’d make the other kind of mess and it was awful*,* I tell you what it’s the hardest job*,* I think it’s one of the hardest jobs anyone can do*,* you’re trying to get through to them what you mean and sometimes you have to shout and then you think “oh I shouldn’t be shouting” but you’re only human*,* but it’s hard*,* especially when they start wetting the bed in the middle of the night and you’d be praying “don’t do it” it was like living a nightmare (Participant 19*,* Wife*,* aged 85)*.

To overcome such problems, some explained that they had to purchase alternative products, which could be costly. One carer even spoke about having to improvise with products that had been designed for dogs to manage leakages. This contributed to a perception of degradation when talking about incontinence:

*Quite often the pads would leak*,* especially in the night*,* I remember a time when my daughter came home with all of these puppy toilet training pads and she said “we can put these on the bed at night just in case” (Participant 11a*,* Husband*,* aged 84)*.

Providing intimate care could also result in changes to relationships. For example, family carers supporting their parents often spoke of a role reversal, using analogies of the person with dementia reverting to a child. Additionally, some spousal carers felt that their relationship had turned into a carer–client relationship, which could cause distress:

*I hated it being stuck in here*,* the smells*,* I could clear up dog’s muck*,* cat’s muck*,* I worked on a farm*,* babies’ nappies*,* nothing on me*,* but try doing it on your wife*,* an adult wife*,* God it would crucify me (Participant 06*,* Husband*,* aged 70)*.

Other issues related to gender and the acceptability of male family carers assisting females with intimate care. This was specifically in relation to adult sons caring for their mothers, who believed it was inappropriate and undignified, instead they relied on professional carers to manage the task.

### Frailty and falls

Family carers explained that during the last year of life, the person with dementia had become increasingly frail and was often restricted to spending most of the day in a bed or chair. Adjustments to the home were usually required to assist with caring for the person with dementia and the use of supportive equipment was necessary:

*It became more and more apparent that mum’s mobility wasn’t really up to it*,* we couldn’t really get her downstairs at that point so we just created a studio bedroom in her bedroom*,* we brought the big chair upstairs and the commode and the hoist and the bed*,* so we could get her out of bed and into a chair and things like that (Participant 11b*,* Daughter*,* aged 55)*.

With increased frailty, falls within the home became more common. Family carers found that managing a fall was physically demanding and often complained that their own bodies had been affected by heavy lifting:

*The following Sunday I was downstairs and there was this great thud and there was mum on the floor by the bed*,* I got her to sitting but I couldn’t get her to do anything else*,* I had to ring [my husband] and got him out of bed but he came and was able to lift her and get her back into bed*,* my back was so bad at that point*,* I couldn’t help (Participant 07*,* Daughter*,* aged 65)*.

Some described using assistive technology, such as Telecare alarms to call for help when the person with dementia had fallen. These were often wireless devices that could be worn as a pendant around the neck or wrist and were linked to a 24-hour monitoring service. However, for some, especially those living in rural areas, the monitoring service was based far away from the home, meaning that on some occasions the person with dementia could be left on the floor for long periods of time.

Ambulance services were also commonly called following a fall. This often led to admissions to hospital which could precipitate into a downward spiral of immobility and incapacity, resulting in lengthy stays away from the home during the last year of life:

*It was five o clock in the morning he got up to go to the toilet I put the lights on and then there was this almighty bang and he fell*,* he smashed his hip*,* he was never able to use his right arm again and his left wasn’t much good actually but I think it was his shoulder that caught a lot of the fall as well*,* so he was in hospital for six weeks and then in a nursing home for another eight weeks so I was going every day for fourteen weeks and anyway we finally got him home*,* I wanted him back home and he wanted to come home (Participant 21*,* Wife*,* aged 89)*.

The extended period of immobility during this person’s hospital and care home stay led to a permanent loss of independence. This made it more difficult for his wife to care for him when he returned home.

### Pain

Family carers highlighted that pain management was a central aspect of end-of-life care at home and a main concern was that the person with dementia was pain free. Consequently, the need for effective pain assessment and control was highlighted as an important issue. Family carers found it difficult to determine if the person with dementia was in pain, as they were often unable to communicate verbally. Some felt this placed people with dementia at a distinct disadvantage when compared with those dying from other conditions:

*We were wondering if she’d had a cancer or something*,* would’ve things been different*,* you know if she could verbalise her pain*,* you know people whose parents have passed away with cancer they seem to have been in a lovely hospice*,* pain relief twenty-four hours a day*,* we got none of that*,* it was frantic phone calls*,* those people can say if they’re in pain*,* we could only say we think she is so it’s very different isn’t it? (Participant 22a*,* Granddaughter*,* aged 22)*

Family carers also reflected on the reticence of healthcare professionals to prescribe anticipatory pain relief, and this was recognised as a barrier to achieving adequate pain control at home:

*The problem was we’d have to phone the district nurses and say “she’s in pain”*,* by the time they would come out she was calm so they said “we can only go with what we’re seeing now” so I’d say “ok so you’re going to go again and then I’ll have to phone you again” and they’d say “yes” and I felt awful pestering them but then in the end [my daughter] was getting so angry and she said “well I’m going to pester” so that’s what was hard at home*,* you kept having to phone to get people to give the pain relief (Participant 22b*,* Daughter*,* aged 55)*.

Having to manage the person’s pain, often at irregular hours, could exacerbate family carer’s stress and anxiety. They often worried about getting the right medication in time and feared being unable to relieve the person’s suffering. This appeared to add to a heightened sense of helplessness.

### Dysphagia

Swallowing difficulties were also noted as a common symptom towards the end-of-life. Many family carers reported that they had not been told that this could occur with some trying to continue with feeding or hydration:

*I felt awful*,* you know trying to get her to drink stuff*,* I think there was only about four weeks between that and her dying*,* I think she was actually just shutting down*,* you know I was trying to force her to drink but she’d drink a bit but then she couldn’t drink anymore and it comes to a point when you just don’t want to be forcing them*,* it’s very difficult because like one of the nurses said you tended to want to keep feeding them but the body knows what it does and doesn’t want*,* so I was trying to shove drinks down her and stuff but I realised she just didn’t want it and just couldn’t take it (Participant 03*,* Daughter*,* aged 54)*.

Family carers explained that discontinuing food and drink was one of the most distressing decisions that they had to make:

*I put a little bit of toast in her mouth and she didn’t swallow and I think I knew then*,* once the swallow goes*,* that’s it now*,* that’s it*,* so then it was just kind of like keeping her mouth moist but I was still trying to give her some juice and then she choked*,* and it was [my daughters] not the nurses that said “she’s aspirating*,* you’ve got to stop” but to actually stop giving someone a drink*,* that really hit you hard (Participant 22b*,* Daughter*,* aged 55)*.

However, as one carer explained, once it had been appropriately explained by a healthcare professional, although upsetting, it was more likely to be accepted, and the correct course of action could be taken:

*The district nurse said to me*,* the day they fitted this driver thing*,* “you no longer give [your wife] any drink or food or anything” and I said “you’re asking me to starve my wife to death”*,* and she said “no*,* I’m asking you not to choke her to death”*,* she couldn’t drink because actually you were choking her*,* she’d be coughing and spluttering*,* anything that was going into her mouth was going straight in her lungs (Participant 06*,* Husband*,* aged 70)*.

### Living with uncertainty

Uncertainty about the dying process was a common theme across the interviews. Family carers explained that their relative’s dementia trajectory was often characterised by slow, incremental decline. While they could generally present a clear and cogent account of the start of the person’s illness, progression of the condition was often described as a gradual process complicated by comorbidity and spread over a long period of time, in many cases over years rather than months. The difficulties in recognising when the person with dementia was dying appeared to be due a lack of knowledge and ambiguous communication with healthcare professionals.

### Lack of knowledge

Family carers stated that they lacked knowledge about the course of dementia as they found it difficult to obtain information. This included information on the progression of the condition, how this affects the person with dementia and what to expect as end-of-life approached. Carers felt that if these changes had been explained to them, they may have been better prepared to manage them:

*If you’re looking after someone with dementia there is always the fear of what’s going to happen next*,* you need someone to tell you “well don’t worry the next stage will be this but we’ll be able to do this and perhaps if you can’t do that then you can do this” I was always in fear of like what’s going to happen next (Participant 26*,* Daughter*,* aged 46)*.

Additionally, carers spoke about having to be proactive when seeking out information about end-of-life issues. In many cases, reporting that healthcare professionals were not forthcoming with advice about potential resources that would have been useful at the end-of-life:

*Now you’re not told*,* no one says “right these are the things you’re entitled to”*,* you know “you can have incontinence pads once the patient is incontinent*,* you can have a little bit of respite”*,* you’re not told any of that (Participant 12*,* Daughter*,* aged 50)*.

Some explained that they had found out about potential resources and support fortuitously, relying on information from friends or other carers:

*I managed to talk to one of my friends whose mum was going through the same and she said “you can get this and you can get that”*,* she said “get onto the council because she doesn’t have to pay council tax” I said “you’re joking” I didn’t know anything*,* it was her who said I could get a carer’s allowance too*,* it was things that I didn’t know and someone else had to tell me (Participant 08*,* Daughter*,* aged 58)*.

This lack of structure or process in ongoing information sharing may have undermined family carers’ capacity to develop a comprehensive understanding of the disease process and the resources available to them.

### Ambiguous communication with healthcare professionals

Being able to predict the end-of-life phase was further complicated by a sense of ambiguity from healthcare professionals. Family carers explained that despite the involvement of a wide range of professionals, many had failed to recognise the end-of-life phase:

*The recognition of disease progression and end-of-life stage from healthcare professionals*,* that was very poor*,* really really poor (Participant 04*,* Daughter*,* aged 48)*.

In some cases where family carers had recognised that the person with dementia was dying, they found that their judgements were ignored by healthcare professionals:

*It became pretty evident that she wasn’t improving with the antibiotics*,* I remember speaking with the district nurse one day*,* I don’t know whether she was trying to gee us along but it was completely an inappropriate thing to say*,* she just turned round and said “oh dementia patients can go on for years like this*,* we might have another ten years” and I remember just looking at her saying “really?” and “oh well we could have another ten years where she’s just in a chair or in a bed”*,* I said “right*,* I’m going to get you some research and you need to go home and read up on vascular dementia because I’m actually quite insulted by what you’re saying” and I asked her to leave at that point (laughs) so it wasn’t a good start and also she kept going on and on and on about constipation causing more confusion and I said “this isn’t constipation*,* she’s deteriorating she is approaching end-of-life” so I got very frustrated (Participant 24*,* Daughter*,* aged 59)*.

In some instances, a failure of healthcare professionals acknowledging that the person with dementia was dying meant that resources were not put in place in a timely manner. In others, it led to healthcare professionals arranging care that focused on prolongation of life rather than on palliation:

*The next thing it was difficult to get her to eat and drink (sighs)*,* then she sort of started this gurgling and I became a bit disturbed about it and rang the 111 and they came out and they suggested hospital and I didn’t really want her to go to hospital*,* anyway the on call doctor persuaded me it was the best thing so she went in on the seventeenth of January and they put her on a drip*,* then she died on the twenty-fourth*,* never really recovering from that and whilst she was in there she didn’t eat or drink either (Participant 17*,* Husband*,* aged 74)*.

In those cases where end-of-life was recognised by healthcare professionals, families then received the appropriate support that enabled the person with dementia to remain at home:

*Within the next couple of days things ramped up we had the district nurse arranged for four visits a day they’d had discussions about Continuing Health Care funding*,* the hoist was abandoned coz it was recognised that it wasn’t appropriate and that actually it was just about comfort*,* making her comfortable in bed (Participant 04*,* Daughter*,* aged 48)*.

Recognising the end-of-life stage helped to coordinate care that aligned with the person’s wish to stay at home. It provided the family time to prepare and ensured that the person with dementia was comfortable.

### Impacts on wellbeing

Family carers explained how supporting a person with dementia at home until the end-of-life could be all-encompassing and they described how it affected their health, both physically and mentally. They described striving to find a balance between their role as a carer and having a life of their own. However, the challenges encountered at the end-of-life meant that this balance was often disrupted, leading carers to becoming isolated and feeling alone.

### A balancing act

Family carers described having to balance the demands of caregiving alongside other responsibilities such as family life and work. Particularly for those who lived with the person with dementia, there was a sense that caring could become all-consuming. This often meant that familiar routines disappeared as the person with dementia became the constant focus of attention. This could result in family carers neglecting their own needs:

*One doctor said “what you’ve done you’ve looked after your husband far too long*,* you shouldn’t have done that because you haven’t looked after yourself*,* all’s you’ve thought about is your husband and you’ve just let yourself get run down”*,* so I come home after a week or so later I was back in [hospital] again and he said “well I expected this because you’ve really got yourself run down and your age is against you as well”*,* I hadn’t looked after myself*,* I lost a lot of weight*,* it seemed to just go from me but you don’t notice*,* the doctor looked at me and said “what you need is sleep”*,* my eyes were black*,* I didn’t realise*,* you don’t*,* you just carry on (Participant 19*,* Wife*,* aged 85)*.

Even for carers who did not live with the person with dementia, supporting them still required a great deal of commitment:

*I mean you’re so busy when you’re caring*,* I don’t think I realised how much time it was taking until she wasn’t there anymore and suddenly we’ve got these days*,* you just don’t realise how much time it takes and we were lucky that we were retired of course*,* otherwise it would be really difficult (Participant 20*,* Niece*,* aged 78)*.

For those who were still in paid employment, maintaining work alongside caring demands often became impossible, with many having to quit their jobs:

*I managed part time up until this time last year and then it was making me ill and it was getting worse and my job was getting more responsible and one day I just went into work (begins to cry) and I couldn’t stop crying……I just said “you know I want to give my notice in”*,* so he was really good he said “no go sick” so I went sick for about a month didn’t I and my husband just said “call it a day”*,* so that’s what I did*,* I left last year because it was just getting too much mentally for me (Participant 18b*,* Daughter*,* aged 55)*.

Family carers who were still of working age when the person with dementia died, believed that being out of work for long periods of time whilst caring had impacted on their prospects and employability:

*Now I’m kind of unemployable because I’ve been out looking after my mum for so many years and we don’t have much of a pension*,* so money becomes precious then (Participant 03*,* Daughter*,* aged 54)*.

Carers also described how their caring responsibilities could impact on other family relationships and interfere with the lives of others:

*I still have young children at home*,* and it would affect them*,* I’m sure it did because there was always something going on with Mum*,* some kind of issue*,* that would interrupt mealtimes or homework and they couldn’t really have their friends over*,* we couldn’t even really go for days out together because there was no one else here to look after her (Participant 12*,* Daughter*,* aged 50)*.

One carer also explained that it had affected her sense of privacy as she had resorted to sharing a bed with her mother in the last few years of her life to keep her safe:

*When she broke her leg the second time I didn’t go back to my bed because she was more wandering about and I thought “I’ll sleep better when I know where she is” and we slept in that double bed but sometimes she would not stop talking*,* quite good conversation and vocabulary*,* proper words and that but she would just not shut up so that was hard as I had no private time to myself (Participant 15*,* Daughter*,* aged 57)*.

Following the interview, this carer continued to reflect on her fourteen-year journey of providing care for her mother. She proclaimed it had prevented her from making and retaining friends and even finding a romantic partner. She added that since her mother’s death she had spent most of her time alone.

### Social isolation and loneliness

As the person with dementia’s health began to decline in the last year of life, family carers found that they became increasingly unable to leave the house. This meant that their daily lives were characterised by a limited freedom to manage their own time, a lack of sponteneity and less opportunity to engage in things that they enjoyed:

*The biggest problem is not being able to go where you wanted*,* I’d think to myself sometimes*,* “you know I’d love a day out on the train” because when I could go where I wanted*,* I’d really enjoy going out on the train but I just couldn’t do it*,* I had to forget about in a way*,* it was a bit of an upset that you couldn’t do what you wanted*,* you know I couldn’t go for a pint*,* couldn’t leave her on her own at night (Participant 09*,* Son*,* aged 63)*.

In some cases, the feeling of restriction was so intense that carers described feelings of being physically “trapped” or “caged”:

*This place became like a cage*,* this place was dreadful*,* it was like being in a cage (Participant 06*,* Husband*,* aged 70)*.

Some also explained that this feeling of entrapment could also be experienced on an intellectual level as the person with dementia was no longer able to communicate in the same way:

*When my mum came to live with me here then I was thinking “now I’m trapped*,* before I could go shopping on my own*,* but now I’ve got my mum in the house all of the time*,* I’m trapped here”*,* it really gets to you because you’re living in this sort of daft world*,* that it sort of it just really sweeps you up*,* I’d have to put podcasts on about intelligent stuff you know*,* scientific things*,* just to keep my brain going (Participant 03*,* Daughter*,* aged 54)*.

Even for those who were able to leave the house with the person with dementia, there was still a sense that other people did not understand their situation, which was linked to feeling stigmatised and isolated, which could impact on the family carers’ self-esteem:

*There were certainly places in [name of city] that I discovered in the time with [my husband] and his illness*,* that there were places where you definitely weren’t welcome it was almost as people didn’t pay a lot of money to come out to have someone like that sitting next to them*,* it’s not what they go out for*,* the isolation*,* the terrible sort of loss of self-esteem that you have*,* I don’t know how*,* I’ve only just experienced it*,* I would certainly build it into something*,* you know*,* building up the esteem of the person whose looking after them*,* I don’t know if that happens to everybody else or that’s just my experience (Participant 01*,* Wife*,* aged 65)*.

Family carers also reported experiencing stigma from family and friends. This was often described as having more of a negative impact with some carers disclosing that it made them feel “hurt” (Participant 25) or “angry” (Participant 08) as people would no longer visit the person with dementia. Furthermore, some family carers reported that the retraction in the person with dementia’s social network had begun much earlier due to feelings of embarrassment following diagnosis, suggesting that this stigma had also been internalised:

*Mum and Dad had always had an active social life*,* but friends trailed off*,* they didn’t know how to deal with it*,* some of it I put down to the early days when mum was embarrassed so she withdrew and dad probably allowed her to withdraw and he couldn’t explain to any friends (Participant 04*,* Daughter*,* aged 48)*.

Family carers often used social media platforms such as Twitter and Facebook to connect with other carers who were in a similar situation. Here they could find information, tips and resources related to caring, allowing them to share their own experiences and insights, which helped others to navigate similar challenges. This made them feel less alone:

*I found that the dementia group was very helpful and made me feel like I wasn’t the only one going through this*,* I must admit*,* there is a community there the Twitter dementia community which is absolutely wonderful*,* if I came up against any problem I would tweet and I would ask advice and much better advice than you would get from anywhere (Participant 25*,* Wife*,* aged 79)*.

This exchange of advice could be empowering for family carers and offer new strategies for managing their caring responsibilities.

## Discussion

This study explored the experiences of bereaved family carers who had provided care to a person with dementia at home in their last year of life. Interviews revealed that for most, this role was emotionally, mentally, and physically overwhelming. Carers were required to complete a range of complex care tasks including symptom assessment and management, medication administration and personal care. Many had to adopt new responsibilities and learn new skills, whilst expressing concern that they had received no formal training or guidance on the practicalities of physical care.

In the absence of information or instruction, it was clear that many learnt through “trial and error,” or in a reactive way after a crisis. Both of which could be distressing for the carer and the person with dementia. Similar findings have been reported in other studies of family carers providing home-based end stage care to people with cancer [34].

Family carers emphasised the importance of learning how to provide “hands on” care for the person with dementia in the home, as well as the psychosocial and relational aspects of care. Many stressed the need for practical “how-to” information, such as instructions on how to administer medications or advice on moving and lifting the person with dementia. Incontinence care was also highlighted as a huge obstacle to caring at home in the last year of life. Incontinence increases the level of dependence and is believed to place a heavier burden on family carers of people with dementia [35]. Consequently, it is often one of the key factors in a family’s decision to seek a transfer to residential settings [36].

A major challenge to continence management is the lack of evidence-based approaches or guidelines [37]. Evidence suggests that there is an over-reliance on “containment” and the use of products such as pads. This can overshadow other critical aspects of continence care such as addressing underlying causes and prevention. However, where continence cannot be maintained, the use of the right products can significantly improve outcomes and preserve the person’s dignity [38]. Many family carers in this study found that they had not been provided with good quality incontinence products, meaning they often had to purchase expensive alternatives. This raises questions about inequity for those who cannot afford to pay. All family carers of people with dementia need support and guidance on how to manage incontinence and obtain appropriate products. However, staff in all settings often lack knowledge and training on how to manage incontinence well [38, 39]. This is an issue that requires urgent attention.

Family carers also described other changes that occurred to the person with dementia in the last few months of life, such as loss of ambulation, pain, and dysphagia. They reported that they lacked basic knowledge about such common end-of-life symptoms or conditions and were not always aware that these were associated with dying, admitting that they had a limited understanding of the prognostic course of dementia.

Recognising when a person is dying is the crucial first step to planning and delivering effective end-of-life care as it allows healthcare professionals to introduce support, discuss preferences and make advance plans [40]. However, despite wishing to care for the person with dementia at home until death, family carers had received little information about what to expect during the end-of-life phase. As a result, many expressed that they had found end-of-life symptoms alarming and difficult to manage. A lack of self-confidence in their own ability to provide adequate care, or simply not knowing what to do or who to contact when the person with dementia’s health deteriorated, could result in home care ceasing due to a belief that the hospital or a care home would be better equipped to manage their symptoms. This supports longitudinal research by [41], who found that family carers of people with dementia felt that a lack of basic information about the progression of the condition left them struggling to adapt to changes and feeling ill-prepared to manage symptoms.

Advance Care Planning (ACP) can provide people with dementia and their family carers with anticipatory guidance about trajectories of decline and information about common developments associated with end stage dementia such as incontinence, weight loss, pneumonia, and falls [5]. ACP also encourages families to think about future care choices and how these may be supported. Despite efforts to increase ACP for people with dementia [42], this does not always happen [43] and families are required to make heath care decisions on behalf of the person with dementia, which they often find difficult [44]. In this study, family carers had to make decisions related to transfers to hospital, nutrition and hydration, and life prolonging treatments. All of which could be highly emotional and impact on whether the person with dementia could stay at home.

Decision aids are effective tools to support decision making among patients and family carers and can improve knowledge and expectations [45]. In recent years, a decision aid has been co-developed with family carers, people with dementia and healthcare professionals to support family carers making decisions on behalf of the person with severe dementia or those towards the end-of-life [46]. The decision aid covers multiple decisions including changes in care (e.g. transitions to hospital or care home); eating and drinking difficulties; everyday well-being for the person with dementia; and healthcare, tests and medication. A feasibility study [47] found that the decision aid can reduce decisional conflict, suggesting that it supported family carers’ confidence and certainty about making decisions. Measures of distress and satisfaction with care were also improved. The researchers concluded that this aid could be distributed by GPs in consultations with family carers, for example, supporting shared decision‐making, which is a priority for the NHS Long Term Plan [48].

While most family carers found it difficult to predict when the person with dementia was close to death, it must also be noted that some did recognise signs which they believed to be indicative of the person with dementia reaching the end-of-life. However, they reported difficulties when trying to convey this information to healthcare professionals and there was often a sense that their knowledge was ignored or not taken seriously. This could make them either question their own judgements or feel like they were not being listened to, compounding the ambiguity and uncertainty that surrounded death and dying.

The epistemic disadvantage described above is significant given that family carers are, by virtue of government policy and campaigns by various organisations [49, 50] entitled to be considered as “equal partners” in decision making. Legislation such as the Care Act [49] in England has been regarded as a major step forward in giving family carers parity of esteem with those they care for and seeks to provide them with greater control and influence over their needs. However, this study demonstrates that in the context of caring for a person with dementia at home at the end-of-life, family carers may feel undermined by healthcare professionals. Palliative care philosophy maintains that the patient and the family together make up the unit of care. Putting this philosophy into effect means continual commitment to ensure that the family carer role is supported and acknowledged by health care professionals.

One intervention which has been shown to improve family carer support in end-of-life care is the Carer Support Needs Assessment Tool (CSNAT) intervention. The CSNAT is an evidence-based, comprehensive measure of carer support needs intended for use as a practice tool in palliative home care [50]. It was designed to be incorporated into a process of assessment and support that is health care practitioner facilitated but carer-led [51]. As such, it enables family carers themselves to identify areas in which they require more support and prioritise those of most importance. This differs from usual practice in which identification of carers needs are professionally led and part of the patient assessment process. While there is limited evidence for its feasibility in dementia caring, when used routinely in other carer groups such as those looking after people with cancer and Motor Neurone Disease [52], it has the potential to normalise support for carers, facilitate delivery of carer-identified support likely to improve carer outcomes, with the potential to locate resources in a more effective manner. Therefore, it may also be an appropriate tool when supporting family carers who are supporting people with dementia.

Many family carers described being socially isolated and admitted to feeling lonely whilst caring. Loneliness and social isolation are increasingly recognised as important societal challenges. Findings from empirical studies indicate increases in loneliness and/or social isolation are independently associated with poorer health [53]. Social isolation is an objective situation characterised by a lack of contact with other people and being disengaged from groups and social activities [54]. Loneliness is considered to be a distinct concept which necessitates a subjective and negative evaluation of the existing status of one’s own social network. It is defined as “the unpleasant experience that occurs when a person’s social network of relations is deficient in some important way, either quantitatively or qualitatively [55].

It is well documented that important life transitions that induce changes in one’s existing or desired social relations and interactions can precipitate the onset of loneliness. Taking on a caring role often constitutes such a transition [56]. Over the course of their caregiving journey, experiences of loneliness were initially linked to the loss of the relationship to the person with dementia, especially in spousal caring relationships. However, as the person’s health continued to deteriorate, loneliness became related to the restrictions that the caregiving situation imposed, as many found that they could not leave the house. In some cases, this sense of restriction was so intense that participants used analogies of feeling imprisoned or being trapped.

Some family carers spoke of the benefits of using the internet to receive support. Through social media platforms, such as Facebook, and other online communities, carers exchanged information and resources across a broad social circle and accessed advice from other carers on day-to-day issues. Since the pandemic, a growing number of older adults have taken up or increased their use of online technology [57]. Studies have found that these digital engagements can foster resilience and help to reduce loneliness [58, 59]. These findings have implications that could potentially inform the co-design of interventions aimed at supporting family carers of people with dementia at home in the last year of life.

### Strengths and limitations

Our findings support and extend the knowledge provided by the limited number of previous studies from the UK and wider international literature on caring for people with dementia at home in the last year of life. While we acknowledge that the data in this study was collected between 2017 and 2018, it illustrates the range of difficult tasks and decisions that are required of family carers when supporting a person with dementia at home in the last year of life. These are challenges that may persist over time and must be considered when guiding efforts to fill gaps in current support systems for dementia care.

As we interviewed bereaved carers rather than those currently supporting a person with dementia, we acknowledge that carers may have had some difficulties with recall. However, bereaved carers were recruited for a number of reasons. Firstly, as illustrated in the literature, it may be difficult to ascertain when a person with dementia is dying. Therefore, it could be more difficult to identify someone who was caring for a person who is dying. Secondly, it was assumed that those who have experienced caring for someone until death would be able to reflect on their whole caregiving journey from start to finish. Thirdly, those who were still actively caring may have been preoccupied with their role.

While we were not able to include extensive diversity in terms of ethnic background, participants did represent a range of ages, socioeconomic status and living circumstances. It was not the aim that the study findings be directly generalisable to other settings, but rather to provide in-depth insight into family carers’ experiences.

## Conclusions

Family carers play a crucial role in delivering care for people with dementia at home in the last year of life. However, family carers’ lack of knowledge and understanding about the prognosis of dementia and end-of-life symptoms, meaning that they feel unprepared and unsupported. As the condition progresses, so too does the family carer’s need for practical information about what to expect at the end-of-life, how to perform physical care tasks, how to use equipment, how to manage symptoms and possible emergencies, and how to access help. Most importantly there should be guidance around realising the enormity of physical care requirements when caring at home in the last year of life.

Helping family carers to support people with dementia at home in the last year of life requires healthcare professionals to recognise family carer expertise and empowering them to share their knowledge and experience, to jointly establish the needs of both the family carer and the person with dementia and how these can be met.

## Data Availability

The data that supports the findings of this study are available at the University of Liverpool Repositories and available on request from the first author. The data are not publicly available due to privacy and ethical restrictions.

## Appendix 1

### Interview schedule

1. Could you start by describing you experience of looking after (insert name of person with dementia).
  - Professional Support
    - What happened when they were diagnosed?
    - What support/care/services did you/they receive when they were diagnosed?
    - What support/care/services did you/they receive as the disease progressed?
    - How did you find out about these services?
    - How was it funded?
  - Informal Support
    - Did you have a good informal support network around you (friends/family)?
    - Who was coming to see you? What help were they giving you?
    - Did you ask for help? How?
    - Was it easy for you to accept help? Why?
2. When did you know that (insert name of person with dementia) was drawing close to the end of their life? What happened during this time?
  - Symptom management
    - What were the PWD’s main symptoms/problems at the end of life?
    - How did you manage these? Who advised you?
    - What were his/her main needs during this time?
    - What types of care did you have to provide?
  - Equipment & Alterations to home
    - What type of equipment did you have? Was it useful? Who provided/funded it?
    - Did you make any alterations to your home?
3. What made you decide that you wanted to keep (insert name of person with dementia) at home during the last phase of their life?
  - What types of support were offered to you and (insert name of person with dementia) during this time?
  - Did you receive any funding?
  - How effective was that support?
  - What was good about it?
  - What was not so good?
4. What were the main challenges of providing care at the end of life?
  - Why do you think that it was difficult for you?
5. What helped you to cope during this time?
  - Who/What was there to support you?
  - Why do you think this was helpful?
6. What else could have been done to support you and (insert name of person with dementia) at the end of their life?
7. Is there anything else that you would like to add that you think might be relevant?

## References

[1] GBD 2019 Dementia Forecasting Collaborators. Estimation of the global prevalence of dementia in 2019 and forecasted prevalence in 2050: an analysis for the global burden of disease study 2019. Lancet Public Health. 2022;7:2. 10.1016/S2468-2667(21)00249-8 PMC8810394 34998485

[2] World Health Organisation. Dementia. 2023. https://www.who.int/news-room/fact-sheets/detail/dementia. Accessed 10 February 2024.

[3] Alzheimer's Society. How many people have dementia in the UK? 2024. Accessed 20 October 2025 https://www.alzheimers.org.uk/blog/how-many-people-have-dementia-uk.

[4] Sampson EL, Candy B, Davis S, Buylova Gola A, Harrington J and King M. Living and dying with advanced dementia: A prospective cohort study of symptoms, service use and care at the end of life. Palliative Medicine. 2018;32;3:668-81. 10.1177/0269216317726443 PMC5987852 28922625

[5] Harrison Dening K, Sampson EL and De Vries K. Advance care planning in dementia: recommendations for healthcare professional. Palliative Care: Research and Treatment 2019;12: 1178224219826579 30833812 10.1177/1178224219826579 PMC6393818

[6] Office for National Statistics. Deaths registered in England and Wales: 2024. 2025. https://www.ons.gov.uk/peoplepopulationandcommunity/birthsdeathsandmarriages/deaths/bulletins/deathsregistrationsummarytables/2024. Accessed 20 Oct 2025.

[7] Etkind SN, Bone AE, Gomes B, Lovell C, Evans J, Higginson IJ and Murtagh FEM. How many people will need palliative care in 2040? Past trends, future projections and implications for services. BMC Medicine.2017;15;1:1-10. 28514961 10.1186/s12916-017-0860-2 PMC5436458

[8] Mataqi M and Aslanpour Z. Factors influencing palliative care in advanced dementia: a systematic review. BMJ Supportive & Palliative Care. 2020;10:145-56. 10.1136/bmjspcare-2018-001692 30944119

[9] Office for National Statistics. Deaths registered in private homes, England and Wales. https://www.ons.gov.uk/peoplepopulationandcommunity/birthsdeathsandmarriages/deaths/articles/deathsinprivatehomesenglandandwales/2020finalandjanuarytojune2021provisional. Accessed 10 February 2025.

[10] Gomes B, Calanzani N, Gysels M, Hall S and Higginson IJ. Heterogeneity and changes in preferences for dying at home: a systematic review. BMC Palliative Care. 2013;12;7:2013 10.1186/1472-684X-12-7 PMC3623898 23414145

[11] Hoare S, Morris ZS and Kelly MP. Do patients want to die at home? A systematic review of the UK literature, focused on missing preferences for place of death. PLoS ONE. 2015;10:11. 10.1371/journal.pone.0142723 PMC4640665 26555077

[12] UK Government. End of Life Care Strategy: promoting high quality care for adults at the end of their life. 2008. https://www.gov.uk/government/publications/end-of-life-care-strategy-promoting-high-quality-care-for-adults-at-the-end-of-their-life. Accessed 10 Feb 2024.

[13] Mogan C, Lloyd-Williams M, Dening Harrison K and Dowrick D. The facilitators and challenges of dying at home with dementia: A narrative synthesis. Palliative Medicine. 2018;32(6):1042–105. 10.1177/0269216318760442 PMC5967035 29781791

[14] Farina N, Page TE, Daley S, Brown A, Bowling A, Basset T, Livingston G, Knapp M, Murray J, and Banerjee S. Factors associated with the quality of life of family carers of people with dementia: A systematic review. Dementia and Alzheimer’s. 2017;5:572-81. 10.1016/j.jalz.2016.12.010 28167069

[15] Alzheimer’s Society. Carers UK’s ‘State of Caring 2021’ Report - Alzheimer’s Society Responds. 2021.https://www.alzheimers.org.uk/news/2021-11-03/carers-uks-state-caring-2021-report-alzheimers-society-responds. Accessed 10 Feb 2024.

[16] Alzheimer's Society. Alzheimer’s Society Briefing – Backbench business debate on value of social care. 2022. https://www.alzheimers.org.uk/sites/default/files/2022-11/Alzheimer%27s%20Society%20Briefing%20November%202022%20-%20BBD%20on%20Value%20of%20Social%20Care.pdf. Accessed 10 Feb 2024.

[17] Alzheimer's Society. What are the costs of dementia care in the UK? 2019.https://www.alzheimers.org.uk/about-us/policy-and-influencing/dementia-scale-impact-numbers. Accessed 10 Feb 2024.

[18] Sarabia-Cobo C and Sarriá E. Satisfaction with caregiving among informal caregivers of elderly people with dementia based on the salutogenic model of health. Appl Nurs Res. 2021 62:151507. 10.1016/j.apnr.2021.151507 34815003

[19] Yuan Q, Zhang Y, Samari E, Jeyagurunathan A, Goveas R, Ng LL, Subramaniam M. Positive aspects of caregiving among informal caregivers of persons with dementia in the Asian context: a qualitative study. BMC Geriatrics. 2023 27;23(1):51. 10.1186/s12877-023-03767-8 PMC9883086 36707781

[20] Quinn C, Toms G, Rippon I, et al. Positive experiences in dementia care-giving: findings from the IDEAL programme. *Ageing and Society*. 2024;44(5):1010-30.

[21] Etters L, Goodall D and Harrison BE. Caregiver burden among dementia patient caregivers: A review of the literature. Journal of the American Academy of Nurse Practitioners. 2008;20;8:423-8. 10.1111/j.1745-7599.2008.00342.x 18786017

[22] Brodaty H and Donkin M. Family caregivers of people with dementia. Dialogues in Clinical Neuroscience. 2009;11;2:217-28. 10.31887/DCNS.2009.11.2/hbrodaty PMC3181916 19585957

[23] Sutcliffe C, Giebel C, Bleijlevens M, Lethin C, Stolt M, Saks M and Soto M. Caring for a Person With Dementia on the Margins of Long-Term Care: A Perspective on Burden From 8 European Countries. J Am Medical Directors Ass. 2017;18;11:967-73. 10.1016/j.jamda.2017.06.004 28733181

[24] Collins RN and Kishita N. Prevalence of depression and burden among informal care-givers of people with dementia: a meta-analysis. Ageing & Society. 2020;40:2355-92.

[25] Shanley C, Russell C, Middleton H and Simpson-Young V. Living through end-stage dementia: The experiences and expressed needs of famly carers. Dementia. 2011;10;3:325-40.

[26] Alzheimer's Research UK. Dementia Statistics Hub. 2022. https://www.alzint.org/about/dementia-facts-figures/dementia-statistics/. Accessed 15 Feb 2024.

[27] Daley S, Murray J, Farina N, Page TE, Brown A, Basset T, Livingston G, Bowling A, Knapp M, Banerjee S. Understanding the quality of life of family carers of people with dementia: Development of a new conceptual framework. Int J Geriatr Psychiatry. 2019;34(1):79-86. 10.1002/gps.4990 PMC6586036 30251443

[28] Mogan C, Harrison Dening K, Dowrick C and Lloyd-Williams M. Health and social care services for people with dementia at home at the end of life: A qualitative study of bereaved informal caregivers’ experiences. Palliative Medicine. 2022;36:976-85. 10.1177/02692163221092624 PMC9174574 35466787

[29] Bentley B, O'Connor M. Conducting research interviews with bereaved family carers: when do we ask? J Palliat Med. 2015;18(3):241-5. 10.1089/jpm.2014.0320 PMC4348368 25517136

[30] Addington-Hall J, McPherson C. After-death interviews with surrogates/bereaved family members: some issues of validity. J Pain Symptom Manage. 2001;22(3):784-90. 10.1016/s0885-3924(01)00330-x 11532591

[31] Braun V, Clarke V. One size fits all? What counts as quality practice in (reflexive) thematic analysis? Qualitative Research in Psychology. 2020;18(3):328–52.

[32] Henderson M, Addington-Hall J and Hotopf M. The willingness of palliative care patients to participate in research. J Pain Symptom Management. 2005;29;2:116-8. 10.1016/j.jpainsymman.2004.12.001 15733802

[33] Gysels M, Shipman C and Higginson IJ. “I will do it if it will help others”: motivations among patients taking part in qualitative studies in palliative care. J Pain Symptom Management. 2008;35;4:347-55. 10.1016/j.jpainsymman.2007.05.012 18243642

[34] Bee PE, Barnes P and Luker KA. A systematic review of informal caregivers' needs in providing home-based end of life care to people with cancer. J Clin Nurs. 2009;18:1379-93. 10.1111/j.1365-2702.2008.02405.x 18624779

[35] Drennan VM, Greenwood N, Cole L, Fader M, Grant R, Rait G and Iliffe S. Conservative interventions for incontinence in people with dementia or cognitive impairment, living at home: a systematic review. BMC Geriatrics. 2012 28;12:77. 10.1186/1471-2318-12-77 PMC3562513 23272951

[36] Luppa M, Luck T, Brähler E and König H. Prediction of institutionalisation in dementia. A systematic review. Dementia and Geriatric Cognitive Disorders.2008 26;1:65-78. 10.1159/000144027 18617737

[37] Burholt V, Davies J, Boyd M and Mullins JM. A research agenda for promoting continence for people living with dementia in the community: Recommendations based on a critical review and expert-by-experience opinion. J Clin Nurs. 2022;31;13:1933-46. 10.1111/jocn.15537 PMC9292568 33091190

[38] Murphy C, De Laine C, Macaulay M, Hislop Lennie K and Fader M. Problems faced by people living at home with dementia and incontinence: causes, consequences and potential solutions. Age and Ageing. 2021;50:944–54. 10.1093/ageing/afaa262 33320926

[39] Drennan VM, Cole L and Iliffe S. A taboo within a stigma? a qualitative study of managing incontinence with people with dementia living at home. BMC Geriatrics. 2011;11:75. 10.1186/1471-2318-11-75 PMC3250935 22081876

[40] National Institute for Health and Care Excellence. NICE Quality Standard 13: End of Life Care for Adults. 2017. https://www.nice.org.uk/guidance/qs13. Accessed 10 Feb 2024.

[41] Moore KJ, Davis S, Gola A, Harrington J, Kupeli N, Vickerstaff V, Jones L and Sampson EL. Experiences of end of life amongst family carers of people with advanced dementia: longitudinal cohort study with mixed methods. BMC Geriatrics. 2017;17:135. 10.1186/s12877-017-0523-3 PMC5496359 28673257

[42] National Institute for Health and Care Excellence. Dementia: assessment, management and support for people living with dementia and their carers. 2018.https://www.nice.org.uk/guidance/ng97. Accessed 19 Feb 2024. 30011160

[43] Saini G, Sampson EL and Davis S. An ethnographic study of strategies to support discussions with family members on end-of-life care for people with advanced dementia in nursing homes. BMC Palliative Care. 2016;15;1. 10.1186/s12904-016-0127-2 PMC4936120 27388766

[44] Davies N, Rait G, Maio L and Iliffe S. Family caregivers' conceptualisation of quality end‐of‐life care for people with dementia: a qualitative study. Palliative Medicine. 2017;31;8:726‐33. 10.1177/0269216316673552 PMC5625846 27815555

[45] Stacey D, Légaré F, Lewis K, Barry MJ, Bennett CL, Eden KB, Holmes-Rovner M, Llewellyn-Thomas H, Lyddiatt A, Thomson R and Trevena L. Decision aids for people facing health treatment or screening decisions. Cochrane Database of Systematic Reviews. 2017;4;4:CD001431. 10.1002/14651858.CD001431.pub5 PMC6478132 28402085

[46] Davies N, Sampson EL, West E, DeSouza T, Manthorpe J, Moore K, Walters K, Harrison Dening K, Ward J and Rait G. A decision aid to support family carers of people living with dementia towards the end-of-life: Coproduction process, outcome and reflections. Health Expectations.2021;24;5:1677-91. 10.1111/hex.13307 PMC8483186 34288291

[47] Davies N, Aker N, Vickerstaff V, Sampson EL and Rait G. A feasibility study of a decision aid to support family carers of people with severe dementia or those towards the end-of-life. Palliative Medicine. 2022;6;9:1432-9. 10.1177/02692163221122379 PMC9596957 36081274

[48] NHS. NHS Long Term Plan. 2019.https://longtermplan.nhs.uk. Accessed 10 Feb 2024.

[49] UK Government. The Care Act. 2014. https://www.legislation.gov.uk/ukpga/2014/23/contents/enacted. Accessed 10 Feb 2024.

[50] Ewing G and Grande G. Development of a Carer Support Needs Assessment Tool (CSNAT) for end-of-life care practice at home: a qualitative study. Palliative Medicine. 2013;27;3:244–56. 10.1177/0269216312440607 22450160

[51] Ewing G, Austin L and Grande G. The role of the Carer Support Needs Assessment Tool in palliative home care: A qualitative study of practitioners’ perspectives of its impact and mechanisms of action. Palliative Med. 2016;30;4:392–400. 10.1177/0269216315596662 26199133

[52] Ewing G, Croke C, Rowland C and Grande G. Suitability and acceptability of the Carer Support Needs Assessment Tool (CSNAT) for the assessment of carers of people with MND: a qualitative study. BMJ Open. 2020;10;12:e039031. 10.1136/bmjopen-2020-039031 PMC7716662 33273047

[53] Holt-Lunstad J, Steptoe A. Social isolation: an underappreciated determinant of physical health. Current Opinion in Psychology. 2022;43:232–7. 10.1016/j.copsyc.2021.07.012 34438331

[54] Taylor HO, Cudjoe TKM, Bu F, Lim MH. The state of loneliness and social isolation research: current knowledge and future directions. BMC Public Health. 2023 1;23(1):1049. 10.1186/s12889-023-15967-3 PMC10233527 37264355

[55] Perlman D and Peplau LA. Towards a social psychology of loneliness. In:Duck S and Gilmour R, editors. Personal Relationships in Disorder, London, Academic Press; 1982. p. 32-56.

[56] Vasileiou K, Barnett J, Barreto M, Vines J, Atkinson M, Lawson S and Wilson M. Experiences of loneliness associated with being an informal caregiver: A qualitative investigation. Frontiers in Psychology. 2017;8;585. 10.3389/fpsyg.2017.00585 PMC5395647 28469589

[57] Newmark R, Allison T, Smith A, Perissinotto C, Kotwal A. I Would be More at a Loss Without It: Technology as a Tool for Resilience for Older Adults During the COVID-19 Pandemic. Innovation and Aging. 2021 17;5(Suppl 1):217–218.

[58] Kamalpour, M., Watson, J.,& Buys, L. How Can Online Communities Support Resilience Factors among Older Adults. International Journal of Human–Computer Interaction. 2020;36(14):1342–53.

[59] Morrow-Howell N, Galucia N, Swinford E. Recovering from the COVID-19 Pandemic: A Focus on Older Adults. Journal of Aging and Social Policy. 2020;32(4-5):526-35. 10.1080/08959420.2020.1759758 32336225

